# The ecomorphology of Caribou ( *Rangifer tarandus*): a geometric morphometric study.

**DOI:** 10.12688/openreseurope.13782.2

**Published:** 2025-04-15

**Authors:** Ana Belén GALÁN LÓPEZ, Ariane Burke, Sandrine Costamagno

**Affiliations:** 1 TRACES, Centre National de la Recherche Scientifique UMR 5608, Toulouse, Cedex 9, 31058, France; 2 Anthropology Department, University of Montreal, Montréal, H3T 1N8, Canada

**Keywords:** Magdalenian, ecomorphology, migration, reindeer, geometric morphometrics.

## Abstract

Paleolithic reindeer ( *Rangifer tarandus*) was a key species for human populations in western and central Europe during much of the Paleolithic period. In Southwestern France, and in particular during the Magdalenian, reindeer frequently figures among the privileged prey of hunter-gatherer groups. However, and despite numerous attempts to reconstruct the migratory behaviour of Paleolithic reindeer, there is no agreement on the degree of mobility of this prey. Modern ethological data indicate that reindeer herds adopt different mobility strategies depending on the type of habitat and the topography of the environment. Through metapodial bones and phalanges cross-sections, our project ‘Reconstructing habitat type and mobility patterns of Rangifer tarandus during the Late Pleistocene in Southwestern France: an ecomorphological study’ (Emorph) quantifies the link between habitat type, mobility, bone density and morphology using computer tomography (CT) and geometric morphometry (GMM). Based initially on the study of extant caribou populations with distinct migratory behaviours, the results obtained could be applied to several Magdalenian assemblages from southwestern France in the future, with the aim of reconstructing the mobility of these tardiglacial reindeer.

## Introduction

The study of reindeer and its role in Upper Palaeolithic subsistence systems (especially during the Magdalenian period) has produced a vast literature ( Aaris-Sørensen *et al.,* 2007; Bahn, 1977; Banks *et al*., 2008; Bouchud, 1954; Bouchud, 1966; Bouchud *et al*., 1953; Costamagno *et al*., 2016; Delpech, 1983; Delpech, 1987; Deplano, 1994; Enloe, 2001; Fontana, 2000; Gordon, 1988a; Gordon, 1988b; Gordon, 1990; Kuntz, 2011; Kuntz & Costamagno, 2011; Lacorre, 1956; Morin, 2008; Spiess, 1979; Weinstock, 2000a; Weinstock, 2000b; Weladji *et al*., 2002; White, 1980). According to several authors (e.g., Driver, 1990; Heard, 1997; Kuntz, 2011; Kuntz & Costamagno, 2011) reindeer mobility patterns are essential to understanding the hunting strategies and mobility of human groups during the Late Pleistocene.

Because of the high frequency of reindeer remains in French sites, it is suggested that human populations were highly dependent on this animal resource ( Kuntz & Costamagno, 2011). In the South of France ( Costamagno, 1999), reindeer was exploited during the whole year. The Magdalenian (18,000–14,000 cal BP) has even been associated with the specialised hunting of reindeer (e.g., Mellars, 2004). But the predominance of reindeer remains in some archaeological sites could simply reflect their local abundance rather than selective hunting strategies ( Costamagno, 1999; Costamagno & Mateos, 2007; Costamagno *et al*., 2016; Fontana & Chauvière, 2018; Grayson & Delpech, 2002).

During the past few decades, there have been several largely unsuccessful attempts to reconstruct the mobility patterns of reindeer. Different hypotheses have been proposed for the Magdalenian period (18,000–14,000 cal BP) in Southwestern France, for example. Early hypotheses proposed that reindeer migrated relatively long distances on a north-south axis, from winter ranges in the Perigord and the Quercy to summer ranges in the Pyrenees and were either followed or intercepted by human hunters along migration routes ( Bahn, 1977; Lacorre, 1956; Gordon, 1988a; Gordon, 1988b; Gordon, 1990). Seasonal data ( Kuntz, 2011) does not support the existence of large-scale migrations on a North-South axis, however. A second hypothesis, based on osteometric and dental data as well as seasonality studies, suggests relatively short-distance migrations, from the Perigord eastwards to the Massif Central ( Delpech, 1983; Delpech, 1987; Kuntz & Costamagno, 2011). Other authors, using antler and tooth eruption evidence, propose that herds remained all year-round in the Perigord ( Bouchud, 1954; Bouchud *et al.,* 1953; Deplano, 1994; Fontana, 2000; Fontana, 2017; Fontana & Chauvière, 2018). Moreover, it is necessary to consider the possibility that patterns of reindeer mobility evolved during the Late Glacial under the selective pressure of significant climatic changes ( Costamagno *et al*., 2016; Delpech, 1983; Delpech, 1987).

In order to go beyond conventional archaeozoological analyses, we propose an actualistic approach from an ecomorphological perspective to address this question. Modern ethological data indicate that reindeer populations adopt different mobility strategies correlated to habitat type and to population density. Thus, long distance migrations tend to take place in tundra and steppe habitats ( Goddard, 2009; Miller, 1990; Schaefer *et al.,* 2000; Seip & McLellan, 2010) while animals living in or near the forest tend to travel shorter distances comparatively with their tundra counterparts ( Thomas & Gray, 2002) except when population densities increase, driving them to migrate further ( Hinkes *et al.,* 2005). Ecomorphology studies the relationship between the functional design of organisms and the environment ( Wainwright, 1991). Several studies have successfully demonstrated that it is possible to use ecomorphology to study past adaptations, even when the taxa involved are quite different from their modern descendants ( Bignon *et al.,* 2005; Bishop, 1999; Curran, 2009; Curran, 2012; DeGusta & Vrba, 2003; DeGusta & Vrba, 2005a; DeGusta & Vrba, 2005b; Kappelman, 1988; Kappelman, 1991; Kappelman *et al.,* 1997; Plummer & Bishop, 1994; Plummer *et al.*, 2008; Scott *et al.,* 1999; Van Asperen, 2010).

Given the importance of reindeer for hunter-gatherer groups during the Last Glacial period in western and central Europe ( Costamagno *et al.,* 2016), the knowledge of their mobility patterns is the key for a better understanding of Palaeolithic hunter-gather lifeways. The purpose of this study is to create a referential framework to study ecomorphological differences between animals from different biomes and with different mobility patterns with the aim of applying this knowledge towards the reconstruction of past reindeer mobility.

### Extant Caribou in Canada: general description and origins

Caribou ( *Rangifer tarandus*), or reindeer as they are known in Eurasia, are medium-sized members of the Cervidae family. In Canada, this species is distributed across boreal, montane, and arctic environments in most Canadian provinces and all territories. Over this broad area, caribou exhibit a huge variability in ecology, genetics, behaviour and morphology ( COSEWIC, 2015). Antlers occur in both sexes, and they are well-adapted to cold environments with dense pelage, large fat stores, a counter-current heat exchange to reduce loss of heat in respiration, and an ability to reduce energy expenditure in the winter by decreasing metabolism. They rut in late autumn and calve in late spring and early summer, the exact timing varying with latitude and environmental and physical conditions ( COSEWIC, 2015).

Caribou is a chionophile species. Their broad hooves and dew claws act as efficient paddles or as ice picks when navigating steep, rocky, and icy mountainsides ( Miller, 1990; Seip & McLellan, 2010) and make walking through the snow easier ( Taillön, 2013). Peary caribou, in particular, have relatively large hooves, particularly useful for breaking up hard snow ( Gunn, 2010). Caribou (specially mountain and tundra caribou) are excellent swimmers, able to cross rivers and lakes ( Taillön, 2013) during their travels. They walk while feeding and moving between feeding areas. Fast walk often occurs during migration or periods of alertness. They can reach a speed of between three to 11km/h ( Miller, 1990).

All caribou are considered to belong to the same species, but they are classified in several subspecies: *Rangifer tarandus groenlandicus* (Barren-Ground caribou), *Rangifer tarandus granti* (Alaskan Barren-Ground caribou), *Rangifer tarandus caribou* (Eastern caribou/Migratory woodland caribou, mountain caribou and boreal caribou/forest-dwelling caribou) and *Rangifer tarandus pearyi* (Peary caribou) ( Miller, 1990). All of these species are present in this study.

The oldest caribou fossils are found in Eastern Beringia (present-day Alaska and the Yukon) and dated to 1.6 Ma ( Harrington, 1999; Weckworth *et al.,* 2012), indicating a Berigian origin for the genus. Banfield´s taxonomy review (1961) and his morphological analyses indicated the existence of two distinct groups: tundra and forest caribou, with three extant subspecies in western North America. Genetic evaluations ( Flagstad & Røed, 2003) have generally supported the ice age isolation and subsequent divergence of *Rangifer tarandus granti* and *Rangifer tarandus groenlandicus* from *Rangifer tarandus caribou.* This split is confirmed by McDevitt *et al.* (2009). In western Canada, federal classification of woodland caribou ( *Rangifer tarandus caribou*) further divides them into four ecotypes (northern, central, southern and boreal) based upon geography and life history characteristics ( Weckworth *et al.,* 2012).

According to mitochondrial DNA (mtDNA) sequences, caribou in North America evolved from two lineages that differentiated in isolation during the last glaciation: the Berigian-Eurasian and the North American lineages, each of which is named for their ancestral sources in two presumed and separate Pleistocene refugia ( COSEWIC, 2015). Caribou from the southern refugium comprise the North American lineage (NAL). With the retreat of the ice sheets, they spread west across the boreal region and north into the Rocky Mountains. The caribou that were isolated north of the ice sheets comprise the Beringian-Eurasian Lineage (BEL). They spread south from the Beringian refugium in present-day Yukon, north into the Arctic Islands and eastwards. In this manner, those of the NAL are the ancestors of the current *Rangifer tarandus caribou* while BEL caribou are the ancestors of modern *Rangifer tarandus groenlandicus, Rangifer tarandus granti* and *Rangifer tarandus pearyi.* The southern clade supposedly evolved south of the continental ice sheet, while the northern clade was in a glacial refugium in Alaska and adjacent Arctic Canada. Caribou populations that contained unique southern gene types are found in Ontario and Newfoundland. In contrast, exclusively northern types occurred in some Yukon herds and in some forest-tundra and tundra populations of barren-ground caribou ( *Rangifer tarandus groenlandicus*) in northern Canada. Most woodland caribou populations in the mountains of southern British Columbia, Alberta and in the boreal forest and taiga across Canada are a mixture of the two clades ( Cavedon *et al.,* 2019; COSEWIC, 2002; McDevitt *et al.,* 2009).

### Habitat, movement, and locomotion

Several classification systems have been proposed for different purposes (behaviour, morphological differences, ecology…). However, the ‘ecotype’ approach, whereby a population or group of populations is associated with a particular set of environmental conditions is adopted in conservation ecology ( COSEWIC, 2011). Ecotypes classify caribou herds according to different life-history strategies and ecological conditions ( COSEWIC, 2011). Although every provincial Government uses their own ecotype classification, three large ecotypes are recognized across Canada: migratory tundra, boreal forest, and mountain ecotypes ( Seip & McLellan, 2010). Some ecologists consider Peary caribou as a fourth ecotype, separate from the migratory tundra ecotype ( Taillön, 2013). Populations from these four ecotypes have been included in our study.

Migratory Tundra caribou include Barren Ground ( *Rangifer tarandus groenlandicus)* and Grant’s caribou ( *Rangifer tarandus granti)*. Barren Ground caribou ( *Rangifer tarandus groenlandicus*), are the caribou *par excellence* ( Gunn, 2010), big herds containing hundreds of thousands of individuals migrate long distances across the tundra. Their range extends from Alaska to Western Greenland and is continuous across northern continental mainland Canada ( COSEWIC, 2016) from Northwestern Yukon to Baffin Island. They differ in size, body proportions, pelage, and behaviour from other caribou subspecies. They undertake long distances in their annual migration, between 800 and 5055 km, depending on the herd and the year ( COSEWIC, 2016; Fancy *et al.,* 1989; Ferguson & Messier, 2000; Joly *et al.,* 2019; Saher & Schmiegelow, 2005) and travel back and forth between northern tundra calving grounds and summer ranges, until fall when they move to winter ranges in the boreal forest. Their habitats are varied, therefore, from prostrate dwarf shrub tundra and upright shrub tundra in the summer to boreal forest in the winter. High Arctic caribou, however, remain on the tundra during all seasons ( Hummel & Ray, 2010) Much of the area comprising the summer range is wet in summer, and lichens are often the most typical plant cover ( Gunn, 2010), being the main source of fodder for caribou ( Taillön, 2013).

Peary Caribou ( *Rangifer tarandus pearyi*) is endemic to the Arctic Archipelago of Canada (except Baffin Island) and is genetically distinct from other caribou in Canada ( COSEWIC, 2015). Their habitat is treeless arctic tundra across the entire annual range ( Bergerud, 2000; Geist, 1998; Gunn, 2010; Taillön, 2013). Peary caribou herds are relatively small, ranging from tens to hundreds of individuals, very different from other tundra dwelling populations. Their seasonal migrations often occur between islands, involving crossing the sea ice or swimming ( COSEWIC, 2011; Jenkins *et al.*, 2011; Miller, 1990; Miller, 2002; Miller & Gunn, 1978). During severe winters, they may make even longer movements to the mainland ( COSEWIC, 2002; Miller, 1990) and erratic large-scale movements among islands are sometimes recorded ( COSEWIC, 2015). Peary caribou movements are shorter compared with Barren-Ground or Eastern Caribou (GRH/LRH), with seasonal migrations up to 500 km ( Geist, 1998; Miller, 1991). They spend their annual cycle on one island or moving between two or more islands. For example, seasonal migrations within the Prince of Wales-Somerset-Boothia complex (Canadian Arctic Archipelago) range between 300–500 km, though longer movements have been recorded ( COSEWIC, 2004).

Boreal forest caribou or woodland caribou ( *Rangifer tarandus caribou*) are genetically, geographically, behaviourally, and morphologically distinct from their migratory tundra counterparts. Woodland caribou live year-round in small groups south of the treeline; they dwell in boreal and open taiga forests, with two main, well-defined seasons: a short summer, wet and slightly humid, followed by a long, cold, and dry winter ( Taillön, 2013). Unlike Barren-ground (tundra) caribou, boreal forest caribou migrate short distances between calving, summer, and winter ranges ( Miller, 2003; Nuttal, 2005), often between 80–100 km, ( Schaefer, 2010; Seip & McLellan, 2010; Thomas & Gray, 2002) or 50–150 km, depending on the population ( Mallory & Hillis, 1998). Boreal Forest (aka Woodland) caribou live all year round in northern coniferous forests and peatlands ( Schaefer, 2010). When population density increases, they increase their range, migrating longer distances; they move across landscape seasonally foraging, and to avoid predators in the calving season ( Thomas & Gray, 2002; Ferguson & Elike, 2004).

Eastern Migratory Caribou or Migratory Woodland caribou ( *Rangifer tarandus caribou*) are divided into the George River Herd (GRH) and Leaf River Herd (LRH), which occur in open-tundra and boreal habitats in northern Labrador and Québec ( Bergerud *et al.,* 2008; COSEWIC, 2017). Although they belong to the woodland caribou subspecies, they behave like Barren-Ground caribou and perform long migrations between northern Québec and Labrador, and the boreal forests of southern Québec in winter ( COSEWIC, 2011; COSEWIC, 2017), with migration distances ranging between 1120 up to 1770 km on average per year ( Leblond *et al.,* 2016)

Mountain caribou ( *Rangifer tarandus caribou*) are both migratory and sedentary ( COSEWIC, 2017). Mainly present in Alberta and British Columbia they inhabit a range of habitats including old-growth forests, lush alpine meadows, barren alpine tundra and glaciers and display a correspondingly broad diversity of habitat use patterns ( Seip & McLellan, 2010). They undertake altitudinal migrations in spring and autumn and tend to calve at upper elevations ( Cavedon *et al*., 2019; Edmonds, 1988; Hegel & Russel, 2013; Saher & Schmiegelow, 2005; Stuart-Smith *et al.,* 1997; Weaver, 2006), although some populations may make four migrations each year. Herds that calve at higher altitudes can move as much as 200 km between seasonal ranges or even more ( COSEWIC, 2011). Other herds perform shorter movements, between 10 to 40 km to their calving grounds ( Cumming & Beange, 1987; Saher & Schmiegelow, 2005; Weaver, 2006). Seasonal range shifts also occur in response to snowfall conditions affecting forage availability and to avoid predation ( Heard & Vagt, 1998; Taillön, 2013). By undertaking altitudinal migrations, Mountain caribou experience a climatic and ecological change similar to that experienced by migratory tundra caribou moving north or south during their long migrations ( Seip & McLellan, 2010). Mountain caribou use a variety of winter habitats, which include wind swept alpine ridges, subalpine forest, and low-elevation forest ( Seip & McLellan, 2010).

Biologists generally distinguish between two mobility patterns for caribou: migratory and sedentary ( Festa-Bianchet *et al.,* 2011). Migratory reindeer travel more than 200 km, often undertaking seasonal migrations in the order of thousands of kilometres, sedentary caribou travel distances of less than 200 km ( Wittmer *et al.,* 2006). This means that Mountain and Woodland caribou are considered sedentary, whilst Peary, Barrenland, and Grant’s caribou, and Eastern Woodland caribou, are considered migratory.

## Methods

### Sample

For this research we compiled a sample of 60 metacarpals, 70 metatarsals, and 117 proximal phalanges, from the four subspecies of caribou described above ( Table 1). The samples come from caribou herds from mountain, boreal forest, and tundra habitats and includes both migratory and sedentary mobility patterns (all of them from British Columbia, Québec, Labrador, Alberta, Nunavut, and Alaska). Due to the fact that most sedentary populations are classified as “Endangered”, the acquisition of samples from these populations was more difficult (since they are barely extant in museums, it was only possible to acquire them through samples sent by biologists acquired when a caribou died in a traffic accident or was found dead in the forest), resulting in uneven sample sizes.

**Table 1.** Reference sample included in the present study and grouped according to subspecies, habitat, and mobility pattern.

	Subspecies		Habitat		Mobility pattern	
**Metacarpal**	*Rangifer tarandus* *caribou*	30	Mountain	12	Sedentary	14
	*Rangifer tarandus groenlandicus*	6	Boreal Forest	7		
	*Rangifer tarandus granti*	5	Tundra	41	Migratory	46
	*Rangifer tarandus pearyi*	19				
**Metatarsal**	*Rangifer tarandus* *caribou*	40	Mountain	17	Sedentary	11
	*Rangifer tarandus groenlandicus*	2	Boreal Forest	9		
	*Rangifer tarandus granti*	6	Tundra	44	Migratory	59
	*Rangifer tarandus pearyi*	22				
**Proximal phalange**	*Rangifer tarandus* *caribou*	82	Mountain	42	Sedentary	36
	*Rangifer tarandus groenlandicus*	13	Boreal Forest	21		
	*Rangifer tarandus granti*	11	Tundra	54	Migratory	81
	*Rangifer tarandus pearyi*	11				

Data about migration distances and habitat of the samples are based on sampling location and data from the literature ( Cavedon *et al.,* 2019; COSEWIC, 2004; COSEWIC, 2011; COSEWIC, 2015; COSEWIC, 2016; COSEWIC, 2017; Cumming & Beange, 1987; Edmonds, 1988; Fancy *et al.,* 1989; Ferguson & Messier, 2000; Festa-Bianchet *et al.,* 2011; Geist, 1998; Gunn, 2010; Heard & Vagt, 1998; Hummel & Ray, 2010; Jenkins *et al.,* 2011; Joly *et al.,* 2019; Mallory & Hillis, 1998; Miller, 2003; Miller, 1990; Miller, 2002; Nuttal, 2005; Saher & Schmiegelow, 2005; Schaefer, 2010; Seip & McLellan, 2010; Stuart-Smith *et al.,* 1997; Taillön, 2013; Thomas & Gray, 2002; Weaver, 2006) and information provided by biologists. Biologists from the Ministry of Forests, Lands, Natural Resource Operations and Rural Development, British Columbia, provided mountain caribou samples and related information about herds (type of herd, sex, and location). Peary caribou, Barren-Ground, and Grant caribou specimens were collected from Canadian Museum of Nature (Ottawa), which houses one of the largest osteological collections in Canada. Eastern migratory and woodland caribou were obtained thanks to biologists from the Forest and Wildlife branch of the Québec Government, and from zooarchaeological labs at Laval University and the University of Montréal.

Metapodial bones and first phalanges are included in this study. Because artiodactyls have evolved to be efficient runners, using an unguligrade foot posture that minimizes the total area in contact with the substrate ( Köhler, 1993; Reese, 2015) podial elements have been used in a number of morphometric studies assessing body size, locomotor behaviour, and habitat preference ( Plummer & Bishop, 1994; Purdue, 1987; Reese, 2015; Scott, 1985; Scott, 2004). In addition, archaeological samples often include these elements, making this study relevant for archaeozoological analysis.

Regarding the methodology followed, bone samples were scanned at the Ecomorphology and Palaeontology lab (Université de Montréal) in order to obtain cross-sectional images using a peripheral quantitative computed tomograph, or CT-scanner (Stratec pQCT) ( Galán López *et al.,* 2021). Both metacarpals and metatarsals were measured at pre-defined scan points corresponding to 20%, 35%, 50%, 65%, and 80% of the total bone length from distal to proximal (following Ruff, 2002). For proximal phalanges, the mid-section (50% section) was scanned. All of the bones were placed in anterior-posterior position and scanned from distal to proximal ends. The scan results were then subjected to a geometric morphological (GMM) analysis.

Measurement error was tested for each section. Ten images per section were digitalised one day and the same ten images the next day. A Procrustes ANOVA was carried out to test the error. Finally, the value of the mean squares of the Procrustes ANOVA of the error was in all the cases lower than the value of the mean squares of the individuals, therefore there is no measurement error in the data.

One-way permutational multivariate analysis of variance (PERMANOVA) and boxplots for cross-sectional geometry (CSG), individuals were also grouped by subspecies according to habitat and mobility pattern, e.g., *Rangifer tarandus caribou*-mountain-sedentary ( Table 2).

**Table 2.** Codes used in the present study.

Code	Meaning
Rtcaribou_MS	Rangifer tarandus caribou Mountain Sedentary
Rtcaribou_BS	Rangifer tarandus caribou BorealForest Sedentary
Rtpeary_TM	Rangifer tarandus peary Tundra Migratory
Rtgroenlandicus_TM	Rangifer tarandus groenlandicus Tundra Migratory
Rtgranti_TM	Rangifer tarandus granti Tundra Migratory
Rtcaribou_MM	Rangifer tarandus caribou Mountain Migratory
Rtcaribou_TM	Rangifer tarandus caribou Tundra Migratory

### Geometric Morphometric analysis

Geometric Morphometric Methods (GMM) use information captured in the form of homologous landmarks to describe each specimen separately. Once cross-sectional images were obtained, landmark data were collected as a curve, reflecting the round shape of the cross sections, using TpsDig2 2.31 ( Rohlf, 2010) software and converted into landmarks with TpsUtil64 1.81 ( Rohlf, 2013) software. Coordinate data were collected several times until an optimum number of landmarks was reached. Curves were resampled to have an equal number of evenly spaced coordinates in each specimen, starting at a homologous landmark (in the middle of the caudal face). Accordingly, in metacarpals 28 landmarks (LMs) were collected for the 20% and 35% sections, 32 LMs for the 50% section, and 35 LMs for the 65% and 80% sections. In metatarsals, we digitized 30 LMs for the 20% section, 32 LMs for the 35% section, and 35 LMs for the 50%, 65%, and 80% sections. Finally, for proximal phalanges, both anterior and posterior, 22 LMs were collected from the mid-section.

As landmarks record shape and size information in the form of Cartesian coordinates, it is possible to compare different elements that are described in a homologous way ( Klingenberg, 2008; ÓHiggins & Johnson, 1988).

Using MorphoJ (1.06d version) software ( Klingenberg, 2011) landmark data per bone and section were analysed based on procrustes superimposition, known as generalized procrustes analysis (GPA). GPA normalizes shape information by the application of superimposition procedures: landmark configurations are translated, rotated, and scaled to remove all information unrelated to shape ( Zelditch *et al.,* 2004).

After GPA, a principal component analysis (PCA) was performed using MorphoJ for data exploration and to reduce the dimensionality of the datasets for the later linear discriminant analysis (LDA). Principal Component scores (PCs) preserve much of the sample variation in a few variables, allowing us to properly represent morphological differences and avoid ‘overfitting’ the discriminant functions in the training set ( Curran, 2012; Kovarovic *et al.,* 2011).

PC scores were then exported to PAST 3.24 software ( Hammer *et al.,* 2001) to carry out further statistical analyses like the one-way Permutational Multivariate Analysis of Variance (PERMANOVA-sequencial Bonferroni correction). PERMANOVA is a geometric partitioning of variation across multivariate data, defined explicitly in the space of a chosen dissimilarity measure, in response to one or more factors in an analysis of variance design. The test performs a classical partitioning, as in analysis of variance (ANOVA), while simultaneously retaining important robust statistical properties of rank-based non-parametric methods. It is a useful statistical tool for the analysis of multivariate data on the basis of Euclidean distances or non-Euclidean embeddable dissimilarity measures ( Anderson, 2017).

The relative weight of the three predictor variables, subspecies ( *Rangifer tarandus granti, groenlandicus, pearyi,* and *caribou*), habitat (mountain, boreal forest, and tundra) and mobility pattern (migratory and sedentary), was statistically tested using a three-way PERMANOVA using the Vegan script in the Adonis package in R-Studio 1.3. 1093 ( R Studio Team, 2020; r-project.org).

Linear discriminant analyses (LDA) were carried out (in PAST 3.24 as well) on the PC scores, per section, in order to discriminate between different mobility types. LDA is an ordination method that discriminates *a priori* established groups by calculating confusion matrices. The Jackknife test was used as a cross-validation method to assess the effectiveness of the resulting models ( Webster & Sheets, 2010). According to Curran's methodology (2012) and because the aim of this study is to determine how well we can discriminate among groups, LDA's were performed iteratively, starting with the first two PCs ( Table 4) as variables, and then adding each subsequent PC to compare cross-validations. To avoid overfitting the analyses, the maximum number of PCs used never exceeded a total cumulative variance of 99%.

LDA comparisons were performed in pairs and permutation tests were computed to test differences between group means. In some cases, when classification could be improved, Log Centroid Size was added as a variable ( Franklin *et al.*, 2008).

Because of the uneven sample sizes across migratory and sedentary ecotypes ( Table 1), which may lead to the largest sample dominating the pattern of variance in the data, the sensitivity of the LDA to the number of specimens in each group was tested using randomisation experiments ( Evin *et al.,* 2013), creating balanced groups by a random selection of a number of specimens in the largest group (migratory) equal to the sample size of smallest group (sedentary).

Finally, in order to visualize shape changes and provide information on morphological changes in their immediate anatomical context, ‘lollipop’ graphs were produced. In this type of graph, landmark positions of the starting shape are indicated by dots (the ‘candy’ of the lollipop) and the shifts of landmarks to the target shape are represented as lines (the ‘lollipop’ stick) ( Klingenberg, 2013).

### Cross-sectional geometry

The cross-sectional geometry of lower limb bone shafts is often analysed to infer physical activity levels ( López & Castillo, 2014; Macintosh *et al*., 2013). This is because of the capacity of bone shafts to adjust their morphology in response to mechanical loading. Using cross sectional images obtained from the CT-scanner, the Fiji 2.1.0 software ( Schindelin *et al.,* 2012) and the BoneJ (v.1) Plugin ( Doube *et al.,* 2010), it is possible to use the geometric properties of the cross section diaphysis to calculate resistance to mechanical stresses ( Lambert *et al.,* 2014).

Here, we are interested in comparing shape results from two different methods (GMM and shape ratio), therefore only the shape variable from this analysis is included. The bending strength index is often considered one of the most informative variables, and it is calculated as the ratio of second moments of area about the orthogonal principal axes (Imax/Imin). Shafts with ratios greater than 1 are linked to greater mechanical stress and thus, physical activity ( Ruff & Hayes, 1983) while scores closer to 1 imply a more sedentary pattern ( Ruff & Hayes, 1983; Stock & Macintosh, 2016). Higher values for the bending strength index are expected in migratory groups of caribou, compared with sedentary groups.

The Shapiro-Wilk normality test was carried out for each numerical variable, and Bartlett and Levene´s tests were used to verify the homogeneity of variances prior to the analysis. Then, in order to test statistical differences between the two mobility groups (sedentary and migratory), t-tests and Wilcoxon’s test (for non-normally distributed variables) were performed. Finally, a series of boxplots were made to see the distribution of significant categories and they were grouped according to subspecies, mobility and habitat. All the tests were performed using R Studio 1.3.1093 and the packages “dplyr” “ggplot2” and “devtools” ( r-project.org).

## Results

### Geometric morphometrics

One-way PERMANOVA (by subspecies according to habitat and mobility pattern as group variable) was carried out on all the sections for both metacarpal and metatarsal PC scores ( Table 3). In metacarpals, the 20% section for Rtpeary_TM (see Table 1 for codes) mainly showed differences with Rtcaribou_BS (p=0,01). Rtgroenlandicus_TM had statistical differences with Rtcaribou_MS (p=0,03), Rtcaribou_BS (p=0,006) and with Rtgranti_TM (p=0,01). Both Rtcaribou_MM and Rt caribou_TM have differences with Rtcaribou_MS (p=0,007 and p=0,01 respectively) and Rtcaribou_BS and Rtgranti_TM (p=0,001) as well. These two sedentary groups showed statistical differences with the rest of the migratory groups. In the 35% section, Rtpeary_TM had differences statistically significant with all the groups, which are especially important with Rtcaribou_MS (p=0,0002), Rtcaribou_MM (p=0,002), and Rtcaribou_TM (p=0,002). In the 50% section, there are no significant statistical differences for any group and in the 65% section we find these differences only between Rtcaribou_TM and Rtcaribou_MS (p=0,003), Rtpeary_TM and Rtcaribou_MM (p=0,02), and Rtgroenlandicus_TM and Rtcaribou_TM (p=0,02). In the 80% section, Rtpeary_TM had differences with all the other groups except Rtcaribou_BS. Rtcaribou_TM, and Rtcaribou_MS (p=0,07) which had significant values.

**Table 3.** PERMANOVA per section according to mobility pattern as group variable.

Bone	Section	Mobility Pattern	*P- value*
Metacarpal	20%	Migratory----Sedentary	0,0002
Metacarpal	35%	Migratory----Sedentary	0,0054
Metacarpal	50%	Migratory---Sedentary	0,0396
Metacarpal	65%	Migratory----Sedentary	0,0369
Metacarpal	80%	Migratory----Sedentary	0,2481
Metatarsal	20%	Migratory----Sedentary	0,0154
Metatarsal	35%	Migratory----Sedentary	0,0023
Metatarsal	50%	Migratory----Sedentary	0,0207
Metatarsal	65%	Migratory----Sedentary	0,0289
Metatarsal	80%	Migratory----Sedentary	0,1966
Prox. Phalanges-anterior-	50%	Migratory----Sedentary	0,0004
Prox. Phalanges-Posterior.	50%	Migratory----Sedentary	0,5396

**Table 4.** Number of principal components (PCs) used in linear discriminant analysis (LDA), per section and bone.

Bone	Section	PCs	PC variance % (accumulated)	% Resubstitution	% Cross-validation
Metacarpal	20%	5	92,52	90%	88,33
Metacarpal	35%	8	95,69	87,93	84,48
Metacarpal	50%	7	95,40	85	83,33
Metacarpal	65%	14	98,36	95,05	81,97
Metacarpal	80%	15	97,79	90,16	75,41
Metatarsal	20%	3	81,85	78,37	75,71
Metatarsal	35%	6	93,42	84,29	81,43
Metatarsal	50%	15	98,81	86,36	77,27
Metatarsal	65%	14	98,81	84,85	74,24
Metatarsal	80%	8	99	86,57	77,61
Proximal. Phalanges-anterior-	50%	18	98,62	88,68	77,36
Proximal. Phalanges-Posterior.	50%	3	66,74	60,94	57,58

For metatarsals, in the 20% section, differences between groups are less obvious. Major differences with the rest of the groups are shown by Rtpeary_TM and Rtgranti_TM (p=0,002). In the 35% section, the main differences are between Rtcaribou_TM and the rest of the groups (except Rtpeary_TM), and between Rtpeary_TM and the other populations. The 50% section presented mainly statistically significant differences between Rtpeary_TM and Rtcarbou_MM (p=0,08), and Rtcaribou_BS(p=0,0007), Rtcaribou_TM, and Rtgranti_TM (p=0,03). In the 65% section, the major differences are between Rtcaribou_BS and Rtcaribou_MM (p=0,04), Rtpeary_TM and Rtgranti_TM (p=0,03), and between Rtpeary_TM and all the groups except Rtcaribou_MS and Rtgroenlandicus_TM. Finally, in the 80% sections all the groups except Rtcaribou_MS had significant differences with Rtpeary_TM.

Then, a three-way PERMANOVA was performed in all the sections for both metapodial bones and phalanges on PC scores as well, in order to assess the three group variables and explore the interaction between them. The subspecies variable got significant values on the 35% (p=0,002) and 80% (p=0,003) metacarpal sections, and all metatarsals and phalanges sections. However, a larger sample is needed to include that variable in further analyses. Habitat was significant (p=0,003) on the 35% and 80% (p=0,03) metatarsal section, 35% metacarpal section (p=0,01) and posterior first phalange section (p=0,005). Mobility pattern was statistically significant on the following sections in metacarpal: 20% (p=0,001), 35% (p=0,007), 65% (p=0,02); in metatarsal: 20% (p=0,01), 35% (p=0,003), 50% (p=0,02) 65% (p=0,03) and in anterior first phalanx (p=0, 001).

Finally, once the dependent variables were tested, mobility pattern emerged as a good predictor and, due to the size of the reference sample, we considered it a suitable predictor for discriminant analyses.

Then, a one-way PERMANOVA was done only on the mobility pattern variable. The results obtained with the one-way PERMANOVA test indicate statistically significant differences between sedentary and migratory groups per almost every bone section, with the exception of the anterior proximal phalanges ( Table 3) and on the proximal (80%) sections in both metacarpals and metatarsals. These differences are greater on distal ends.

Considering all these results, although all the analyses were carried out on all the sections ( Galán López *et al*., 2021), given the best outcomes were produced for distal sections (20%), we are going to focus mainly on this section (for both metacarpals and metatarsals).

Regarding randomization experiments, taking only five PCs for the metacarpals 20% section, the result was very similar to the unbalanced sample: 96,15% (cross-validated 88,46%) of the specimens were well classified (uneven sample: 93,33%-90%). When Log Centroid Size is added, the accuracy is 96,15% (including cross-validation) (uneven sample: 96,67%-93,33%). Randomization experiments were performed in every section, and the results, like in the metacarpals 20% section were very similar between balanced/unbalanced samples. Therefore, sample size did not affect LDA results.

Referring to LDA, when Log Centroid size is incorporated to the analysis, the 35% section on metatarsals showed an improvement in classification accuracy on mobility pattern.

### Metacarpals

**Figure 1.**
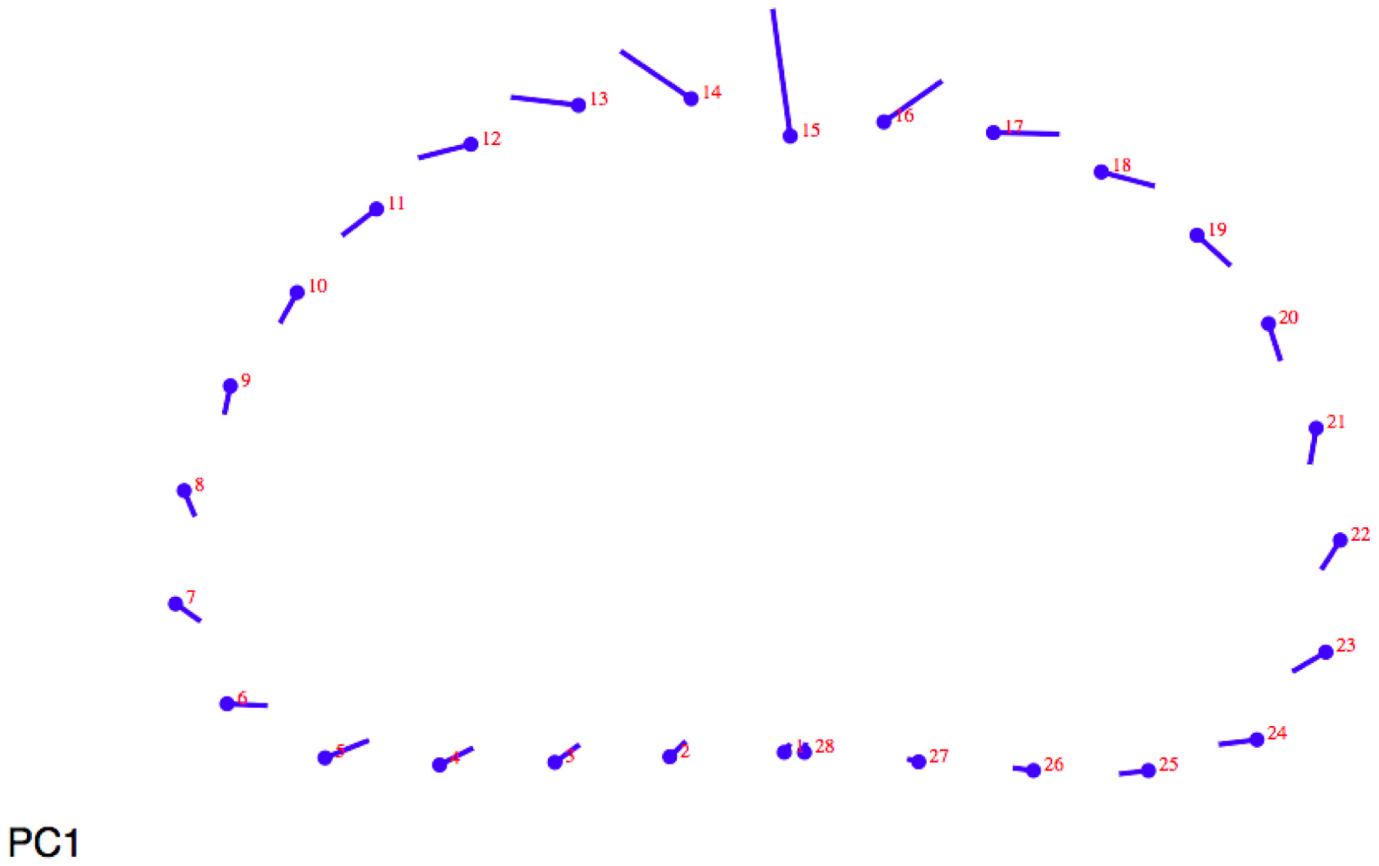
Lollipop graph (20% metacarpal section) visualizing cross-section changes on Principal Component 1 (PC1).

When lollipop graphs are examined in 20% section on metacarpals ( Figure 1) on Principal Component 1 (PC1) showed the biggest differences on the anterior side: migratory individuals have a deeper anterior canal than sedentary ones.

The LDA results ( Table 4) for the 20% section are as follows (including LOOCV values that estimate model performance): five principal components (PC) summarized 92,52% of the total variation ( Table 4 and Table 5). In the first case, 90% of the individuals (leave one out cross-validation, 88,33%) are successfully classified in migratory and sedentary groups. For the 35% section, mobility pattern accounted for 95,69% of the variance (eight PCs) and had a correct classification of 87,93% (leave one out cross-validation 84,48%). The 50% section summarized 95,40% of the variance in first seven PCs for mobility patterns and 85% (83,33% leave one out) of specimens are correctly classified.

**Table 5.** T-tests for each section according to mobility pattern for shape ratio.

t-Test			
	t	Df	*p*
**Metacarpus**			
20%	2.58	54	0.012
35%	0.82	54	0.413
50%	2.23	54	0.029
**Metatarsus**			
20%	0.19	15	0.849
35%	1.77	63	0.079
50%	1.66	63	0.114
65%	0.98	63	0.326
80%	1.47	63	0.143

For the 65% section, 14 principal components accounted 98,36% of the total sample variance. The LDA was able to classify 95,05% of the specimens (leave one out cross-validation 81,97%) correctly according to mobility pattern (migratory/sedentary).

The 80% section showed the lowest classification rates. With 15 principal components that summarized 97,79% of the variance and correctly classifying 90,16% of the specimens (leave one out cross-validation 75,71%) according to mobility pattern.

In view of the results of the PERMANOVA, and in order to test the impact of habitat independently of mobility, a separate LDA was conducted using the 20% section dataset leaving out the sedentary groups (both mountain and boreal forest) individuals and preserving mountain migratory (n= 7) and tundra (n= 41) migratory groups. A new GPA and covariance matrix were carried out to perform a PCA. Then, PCA scores were exported to PAST and LDA was performed iteratively (without exceeding the cumulative 99% variance to avoid overfitting) until obtaining 89,58% (81,25% leave one out cross-validation) of correctly classified cases using 95,53% of the total variance (eight PC scores). However, it should be pointed out that the sample was very unbalanced, with only seven individuals in the mountain migratory group (versus 41 for migratory tundra).

### Metatarsals

**Figure 2.**
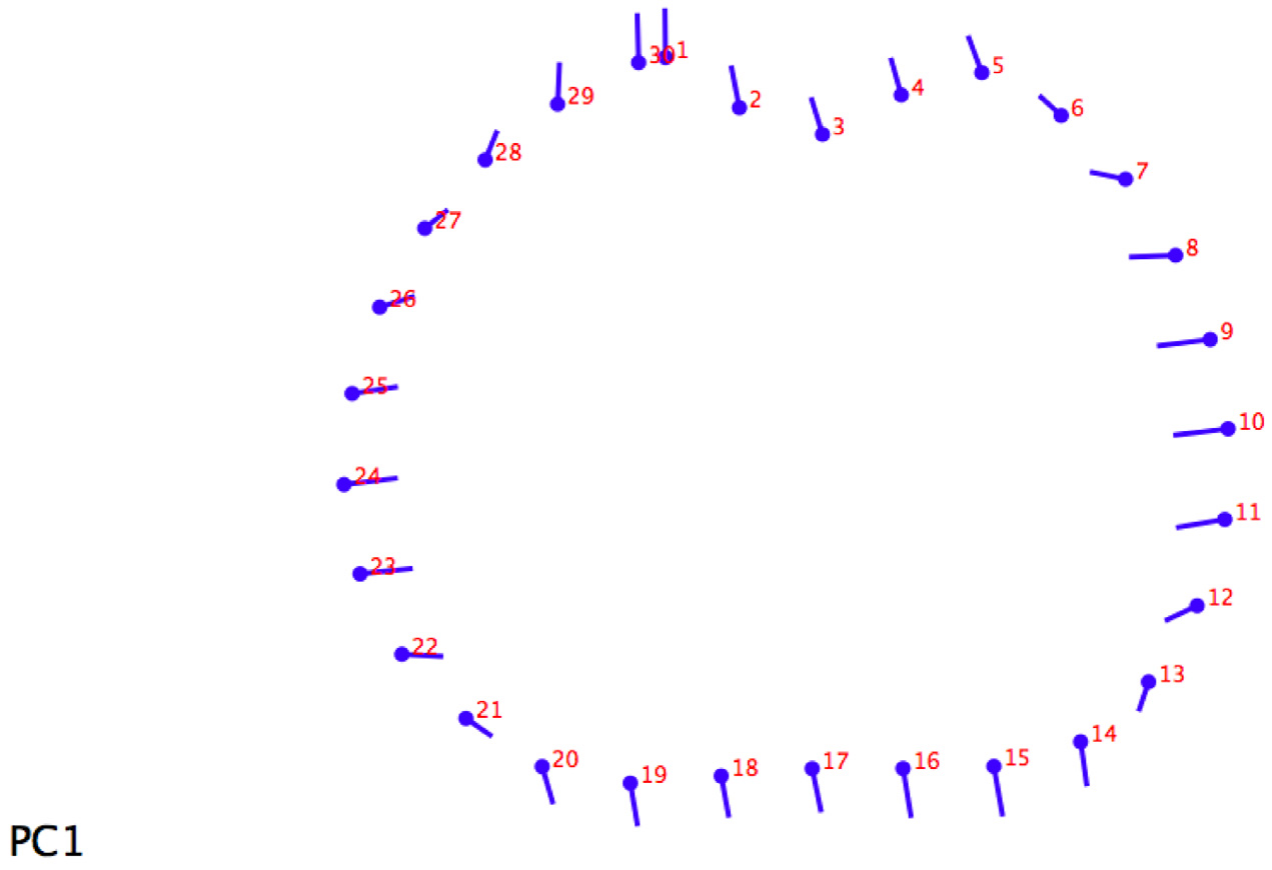
Lollipop graph (20% metatarsal section) visualizing cross-section changes on principal component 1 (PC1).

For the 20% section, Figure 2 shows how differences are distributed homogeneously along the whole section. Migratory individuals have a wider section on their medial-lateral axis than sedentary ones, with the former slenderer than the latter.

The LDA ( Table 4), for the 20% section, used three principal components that accounted 81,85% of the total sample variance; 78,37% (leave one out cross-validation, 75,71%) of the specimens were correctly classified according to mobility pattern.

In the 35% section, six principal components summarized 93,42% of the variance, and the LDA returned a correct classification of 84,29% (leave one out cross-validation, 81,43%) of the specimens into migratory and sedentary groups. For the 50% section using 15 PCs accounting for 98,81% of the total sample variance, 86,36% (leave one out cross-validation, 77,27%) of the specimens are successfully predicted as migratory or sedentary. For the 65% section, using 14 principal components (98,81% of the variance), the LDA successfully classifies 84,85% (leave one out cross-validation, 74,24%) of the specimens by mobility pattern. Finally, for 80% section, using eight PCs accounting for 99% of accumulated total sample variance to the LDA classifies 86,57% (leave one out cross-validation, 77,61%) correctly.

As before, a LDA was performed on the 20% section dataset of metatarsals in MorphoJ leaving out sedentary (mountain and boreal forest) individuals and preserving mountain migratory (n=15) and tundra (n=42) migratory groups. A new GPA and covariance matrix were carried out to perform a PCA. Then, PCA scores were exported to PAST and LDA was performed iteratively. Using a 98,88% of the total variance (17 PC scores), a total of 92,98% (87,72% leave one out cross-validation) were correctly classified.

### Proximal phalanges

It is possible to segregate the anterior proximal phalanges according to mobility pattern (sedentary and migratory) with 18 PCs that summarize 98,62% of the total variance with an accuracy of 88,68% (leave one out 77,36%). Posterior phalanges have low overall classification rates, ranging between 60–50% (including cross-validation rates) ( Table 4).

### Cross-sectional geometry

Finally, a cross-sectional analysis was carried out to analyse geometrical properties. Here, we are going to focus on the shape ratio (Imax/Imin) results, which we suggest are directly linked to mobility and in this case, are also related to our geometric morphometric study. Normality tests (Shapiro-Wilks) showed normal distributions (p>0.05) on metatarsal shape variables and on metacarpals for the 20%, 35%, and 50% sections. The variables from the remaining sections were non-normally distributed. Homogeneity of variances was observed (p>0.05) in all sections of both metacarpals and metatarsals except for 20% (p=0.04) section of metatarsal (Welch test instead of t-test was used because of that).

The t-tests on metacarpals ( Table 5) shows significant differences according to mobility pattern only in the 20% (p=0.01) and 50% (p=0.02) sections. The Wilcoxon test ( Table 6) was also significant on the 65% section (p=0.006). However, t-tests and the Welch test performed on metatarsals produced non-significant values for all sections ( Table 5).

**Table 6.** Wilcoxon tests for metacarpal 65% and 80% sections (non-normal distribution) according to mobility pattern for shape variable.

Wilcoxon-Metacarpus		
	W	*p*
65%	419.5	0.006
80%	370	0.080

Since mobility pattern was only significant on the 20%, 50%, and 65% metacarpal sections ( Table 5), only these were retained for further analysis.

**Figure 3.**
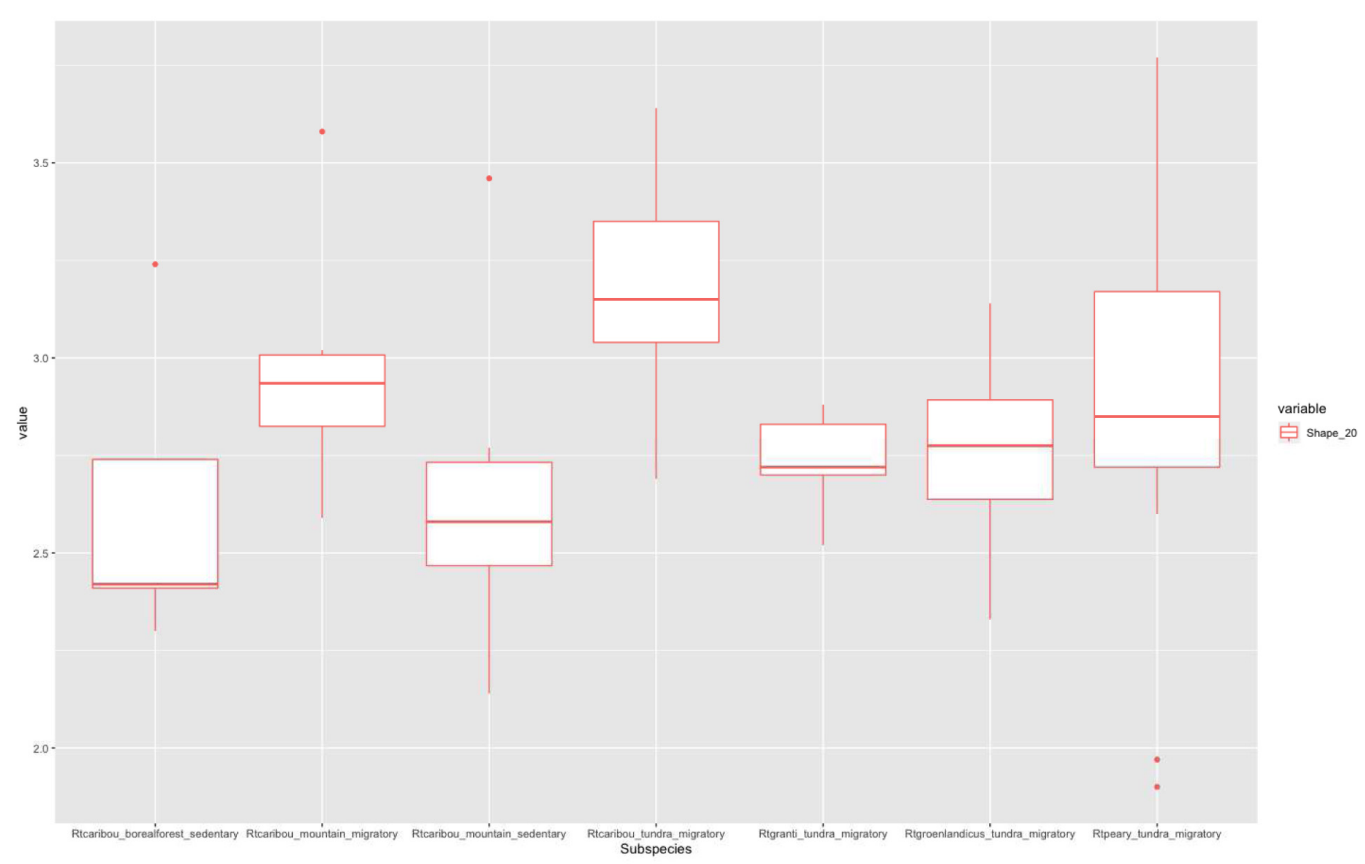
Shape ratio variable (metacarpal 20% section) according to subspecies, habitat, and mobility pattern.

**Figure 4.**
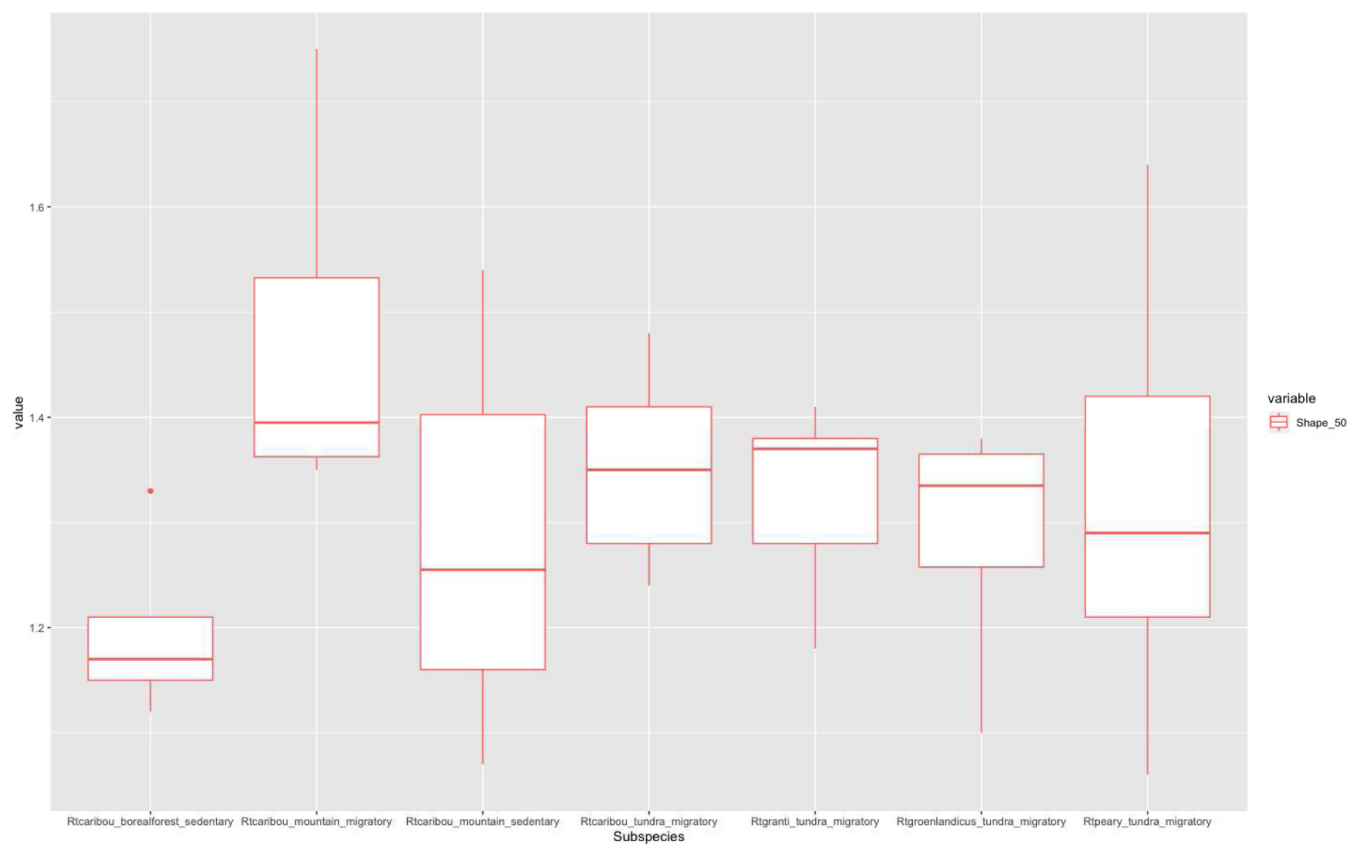
Shape ratio variable (metacarpal 50% section) according to subspecies, habitat, and mobility pattern.

**Figure 5.**
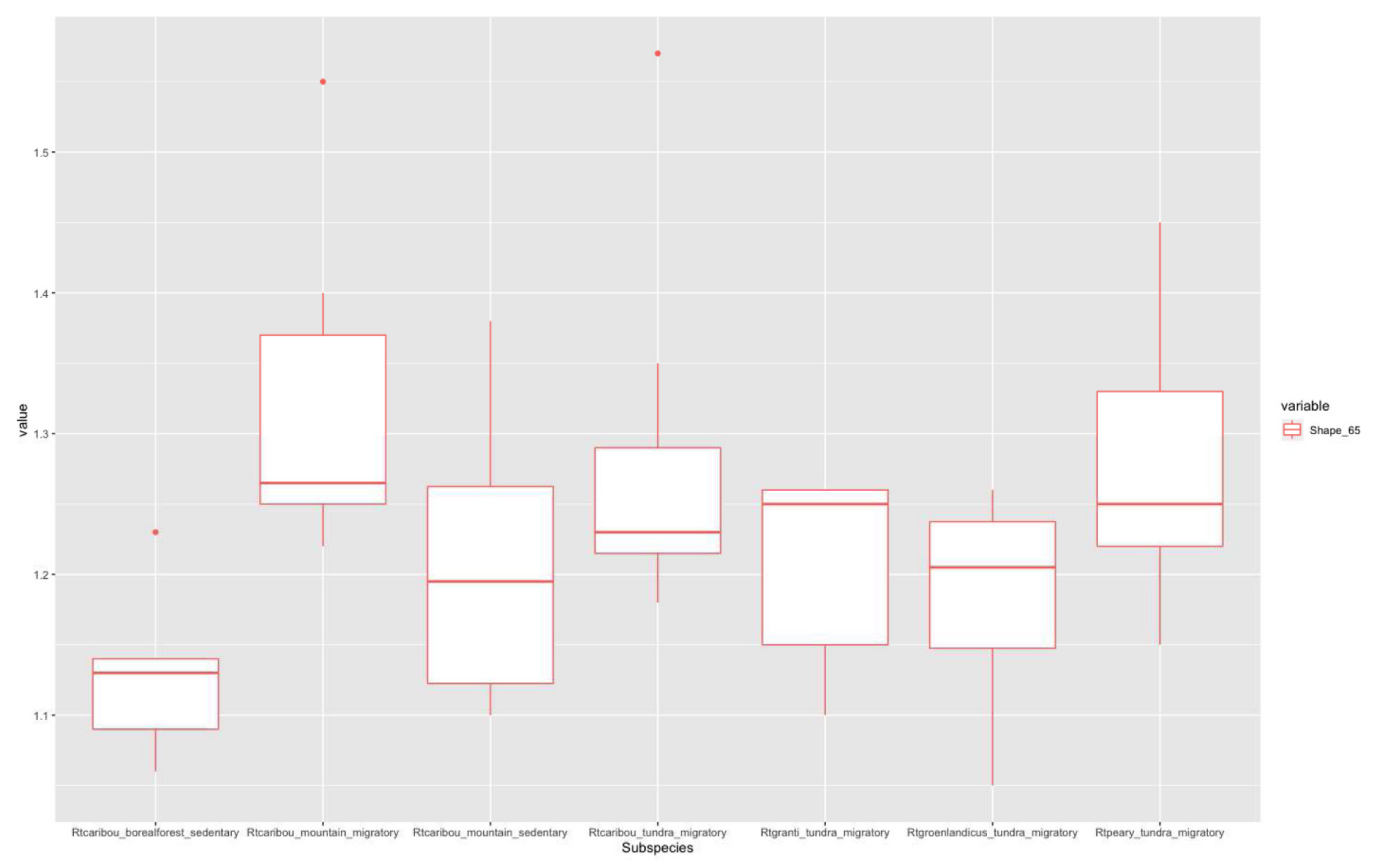
Shape ratio variable (metacarpal 65% section) according to subspecies, habitat, and mobility pattern.

According to Imax/Imin distribution plots, higher values in the 20% section ( Figure 3) correspond to migratory populations. Rtcaribou_borealforest_sedentary and Rtcaribou_mountain_sedentary show a complete overlap. Rtcaribou_tundra_migratory stands out among the rest of the groups. In the 50% section ( Figure 4), the values between the groups are homogeneously distributed, except for R.t.caribou_mountain_migratory, which stands out, and R.t.caribou_boreal forest_sedentary, with the lowest values. In the 65% section ( Figure 5), Rtpeary_tundra_migratory and Rtcaribou_mountain_migratory clearly feature over the rest, with a slight overlap, while Rtcaribou_mountain_sedentary, Rtcaribou_tundra_migratory, Rtgranti_tundra_migratory, and Rtgroenlandicus_tundra_migratory overlap. Rtcaribou_borealforest_sedentary again shows the lowest values.

**Figure 6.**
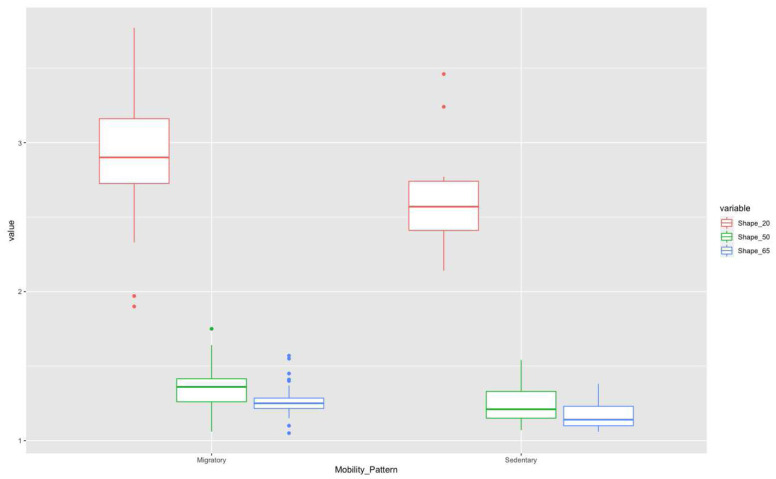
Shape ratio variable (metacarpal 20%, 50%, and 65% sections) according to mobility pattern.

Thus, if we consider only mobility pattern ( Figure 6) in these three sections, in the 20% section, the migratory group is separated from the sedentary one, although with some overlap of some individuals. However, this separation is less evident for the other two sections.

## Discussion

First of all, it is important to notice that the definition of ‘sedentary’ reindeer used in the present study, which is consistent with the biological literature ( Wittmer *et al.,* 2006) is very far from what is meant in an archaeological context. Here, sedentary caribou refers to herds that travel less than 200 km ( Wittmer *et al.,* 2006). For some authors and archaeologists, however, ( Bouchud, 1966; Bouchud *et al.,* 1953; Deplano, 1994; Fontana, 2000; Fontana, 2012; Fontana, 2017), sedentary reindeer behaviour is defined as an annual presence of reindeer in the Périgord area, e.g., at La Madeleine ( Bonnisent, 1993) and layer IX of the Flageolet II ( Deplano, 1994), linked to environmental characteristics ( Fontana, 2000). However, some Woodland caribou and Mountain caribou herds travel very short distances within their annual range ( Hummel & Ray, 2010) and would probably be good proxies for the archaeological model.

The results of the present study (both from geometric morphometrics and shape ratio), show that mobility, which is linked to habitat, affects lower limb bone shape and makes it possible to distinguish between migratory tundra dwellers and sedentary woodland and mountain herds. More sampling and further analyses will be required to test whether or not woodland and mountain populations can be distinguished from one another using these methods.

For the GMM analysis, successful results are produced using distal sections both in metacarpals and metatarsals, especially the 20% section in the metacarpal. The anterior and posterior first phalanges are not suitable for distinguishing mobility patterns according to the low classification rates obtained in this analysis.

Thus, we observe that the best section to apply further analyses on archaeological material is the metacarpal 20% section (higher resubstitution and cross-validation percentages). Differences in shape stand out on the metacarpal (20% section) as well. The main shape differences are found on the anterior face, which is slightly more circular on sedentary individuals and oval (with more of a cranial groove) on migratory ones. Furthermore, migratory individuals have a wider section on their medial-lateral axis than sedentary ones.

Regarding cross-sectional geometry, the only significant differences are in the metacarpal bones, on the 20%, 50% and 65% sections, with the best performance on the 20% section. In this sense, the forelimbs produce better results than the hindlimbs. This makes sense if we consider the fact that thoracic limbs carry 60% of an animal’s static body weight and absorb ground impact as the body is thrown forward by the pelvic structure ( Fletcher, 2019; Zhang *et al.,* 2020). Forelimbs are designed to adopt different locomotor strategies to improve locomotor performance and save energy ( Zhang *et al.,* 2020). Therefore, it is not surprising to find the best evidence of performances on thoracic limbs.

In metacarpals, the transversal section allows us to discriminate migratory and sedentary patterns, with at least a 90% success rate using the distal 20% cross-section. The effectiveness of the 20% section is very important because this part of the bone is often relatively well preserved in archaeological record. In contrast, differences in metatarsals are less pronounced among groups, and they are not as good as metacarpals as indicators of shape variation. However, the metatarsal 20% section showed good results to discriminate among migratory populations. It produced a successful indicator to distinguish between mountain and tundra migratory groups, excluding sedentary individuals from the analysis.

There is correspondence between shape analysis performed using GMM and cross-sectional geometry using shape ratio (Imax/Imin) on the distal 20% section in metacarpals: migratory populations have greater shape ratio values which appear to be linked to higher mobility, and sedentary groups have the lowest scores, which means lower activity patterns. Thus, changes in shape related to mobility and the forces that affect bone are linked to the engineering principle known as “beam theory” ( Ruff & Hayes, 1983). Mobility patterns affect the loads placed on the limb bones during locomotion and influence variation in diaphysis robustness and shape.

On the 20% metacarpal section, greater shape values correspond to Rtcarbou_tundra migratory specimens. However, on the 50% Rtcaribou_mountain_migratory stands out (and also on the 65% section together to Rtpeary_tundra_migratory) over the rest of the groups, probably because of a combination of the altitudinal movement performed by this caribou and differences in the substrate between mountain and tundra populations. The mountain caribou range includes a great habitat variety ( Seip & McLellan, 2010), combining open and close environments: high forest of old-growth cedar and hemlock, vast extensions of pine, spruce and fir forests, alpine meadows, barren alpine tundra, and glaciers which probably creates subtle differences in bone shape or the way that forces or stresses ( Ruff & Hayes, 1983) are affecting the bone, and for that reason we can observe higher values for these mountain individuals.

Sedentary populations that inhabit closed environments, one from Québec and the other from British Columbia, exhibit the same bone morphology, more circular than in their tundra counterparts. The same applies for migratory groups (which all live in open habitats) although some of them belong to different subspecies and migration distances can be variable, all these groups present a common oval shape morphology. Furthermore, considering Imax/Imin ratio results of the 20%, 50%, and 65% sections in metacarpals, there are subtle differences that enable us to identify altitudinal migration (performed by migratory mountain caribou), possibly due to the more varied types of substrate in mountain habitats (as stated above) in contrast to the Peary caribou, which migrate in an open tundra habitat all year round, or Barren Ground and Eastern Migratory Woodland caribou, which move most of the year on open tundra. The 50% and 65% sections reflect that they are more affected by mountain movements which are more altitudinal than longitudinal, which implies greater locomotive effort (reflected in higher values of cross-sectional analyses) than tundra caribou populations which make longitudinal migrations involving longer distances and thousands of kilometres. 

## Conclusion

Geometric morphometrics allows us to distinguish subtle morphological differences and helps us understand the causes of morphological variation in vertebrate species. The present study provides a new and actualistic framework to assess reindeer migration patterns, which are linked to habitat, according to bone shape, discriminating between migratory and sedentary herds.

As Pleistocene environments are not analogous to Holocene ones, it is not possible to infer reindeer mobility patterns from palaeoenvironmental reconstructions. Contemporary caribou herds are found in different environmental settings today and this has allowed us to create a referential framework to study ecomorphological differences between animals from different biomes and mobility patterns. We can also infer habitat type, since those animals which move medium and long distances inhabit mainly open habitats, while those which migrate shorter distances are closed habitat dwellers overall.

Thus, the practical application of this methodology will allow us to reconstruct reindeer movements in future and study how they affected human hunting strategies and understand the precise role of reindeer in their economy during the Magdalenian period.

South-western France is well known for its high density of Upper Palaeolithic archaeological sites ( Castel *et al.,* 2013). The Magdalenian culture (18.000-14.000 cal BP) appears in the region during the Tardiglacial, and the paleoclimatic and environmental variations of the period provide insights into the changes in hunter-gatherer technology, mobility patterns and hunting strategies that occurred during this timeframe ( Langlais *et al.,* 2012). Magdalenian faunal assemblages are frequently dominated by reindeer, one of the best represented taxa in the different climate stages (61% in Late Pleniglacial, 69% in ancient Dryas, 67% in Bølling-Allerød) whose predominance stands out in areas such as the Perigord, Quercy, and Bassin de l’Aude. Mobility patterns of reindeer will have affected the hunting strategies and the mobility of the hunter-gatherer groups that depended upon them. The question of the seasonal behaviour of reindeer remains open ( Kuntz, 2011) and it cannot be solved if we are not able to develop new approaches to this issue. 

In a follow-up study, we propose to use the distal 20% section of the metacarpal to perform this task. In order to establish the degree of mobility of reindeer herds, it is necessary more data provided by a larger sample. So, this is the first step to further analyses. This referential sample and its application to an archaeological record, through the scan of metacarpal 20% distal section and later landmark information, will allow us to be able to infer these two main mobility patterns that have been identified on extant reindeer (caribou), through the use of mainly geometric morphometrics analysis, that have been proven more useful than cross sectional analysis to classify both mobility types.

## Data Availability

Zenodo. EmorphProject: Reconstructing habitat type and mobility patterns of Rangifer tarandus during the Late Pleistocene in Southwestern France: an ecomorphological study https://doi.org/10.5281/zenodo.5105714. ( Galán Lopez *et al.,* 2021) This project contains the following underlying data: CT SCAN EMORPHPROJECT.zip. (Cross-sectional images from metatarsal, metacarpal and first phalanges according to Rangifer tarandus populations.) One-way PERMANOVAS performed in PAST and R respectively. Three-way PERMANOVAS performed in PAST and R respectively. Data are available under the terms of the Creative Commons Attribution 4.0 International license (CC-BY 4.0).
