# Supplementary material for: The ecomorphology of Caribou ( Rangifer tarandus): a geometric morphometric study
Source: Open Res Eur. 2025 Apr 15;1:99. Originally published 2021 Aug 25. [Version 2] doi: 10.12688/openreseurope.13782.2 (PMC12022544; doi:10.12688/openreseurope.13782.2)
Supplement: Peer review history

# Reviewer report: Reviewer response for version 2

**DOI:** 10.21956/openreseurope.21815.r53332

**Published:** 2025-05-03

**Authors:** Andrew Orkney

**Affiliations:** 1 Cornell University, Ithaca, New York, USA

**Summary:**

 The submitting authors wish to develop tools that will help them determine whether Magdalenian remains of Reindeer in France belong to populations that were sedentary or migratory in their behavior. They hence need a morphological proxy from commonly preserved skeletal components that reflects mobility behavior in Reindeer.

 The authors identify contemporary North American Caribou as a suitably closely related lineage that has not been subject to serious changes from domestication, and exhibits a full range of mobility behaviors. The authors collect an expansive set of morphometric measurements from a variety of distal limb bones, although their efforts to do this are partly frustrated by the fact that some populations are endangered.

 The authors undertake Permutation-based ANOVA analyses with a sequential interactive model structure, with mobility tested for first, then habitat and finally subspecies- and report that mobility is frequently associated with a significant difference across populations, and further identity that the distal fifth of the metacarpal is a candidate osteological proxy for mobility, based on a leave-out-out cross validation approach.

 Sexual differences between samples were not apparent in tables, nor was age structure mentioned, which may confound analyses (for example, if migratory populations tend to experience more mortality and the samples are not fully grown, or if random chance means that one population has more males than females and there may be sexual differences in shape.) A previous reviewer identified this potential problem.

 My personal source of doubt originates from the fact that mobility behaviour is highly correlated with Caribou subspecies that are the result of admixture between a Euro-Beringian and Canadian population that are deeply divergent. Differences in osteology may therefore be more likely to reflect differences in ancestry between populations, rather than differences in adaptive responses to mobility. Even if they did reflect adaptation, if those adaptations are specific to a deeply divergent Canadian lineage, analogous adaptations may not have existed in any Magdalenian French population that derives no ancestry from this lineage. The authors' PERMANOVA results recurrently show that subspecies explains a larger portion of the sum of squares variation in osteological properties than either mobility or habitat. I suspect that if the model structure was re-ordered to 'subspecies + habitat + mobility', (which is a more conservative test of the hypothesis), that subspecies would explain a greater portion of the variance and may actually show that mobility does not uniquely explain variance in osteology among North American Caribou populations.

**Is the work clearly and accurately presented and does it engage with the current literature?**

**Response: Partly**

 A previous reviewer pointed out that the data and analysis in the study is focused on osteological differences among North American Caribou populations, while the stated aims are to improve knowledge of behaviors in Magdalenian populations of southern France. I understand why this may confuse some readers, who might expect Reindeer remains from France to be included in at least some capacity.

 I do, however, believe that the authors explain well that western and northern North American Caribou populations and Eurasian ice age Reindeer derive from a hypothesized Euro-Beringian population, while European populations have experienced widespread domestic influences, which means that some North American specimens may be more appropriate representations of ice age Europe than are contemporary Eurasian populations. There are some reasons that this might not be true, due to existing population structure and admixture in the North American Caribou population, but I have reserved this discussion for my comments on study design, statistical analyses and conclusions.

 I note that the authors discuss the semantic differences between migratory and sedentary behaviors described in literature about the ice age and contemporary Caribou populations, and that a migratory-sedentary dichotomy is not absolute- populations switch strategies as needed. I think it is useful that this is mentioned early in the authors’ discussion, but it would be even better if this were mentioned more explicitly in the introduction or methods in order to justify the authors’ choice of study design.

 I discovered two examples of text which appears to have been copied and pasted from previous work by Anderson 2014 and Anderson 2017. The clause ‘while simultaneously retaining important robust statistical properties of rank-based nonparametric multivariate methods,’ was identical to a sentence in Anderson 2014 [Ref:4], implying that the larger statement it was a part of had been copied and pasted, with a subsequent rearrangement to produce ‘original’ text. I wish to discourage this style of writing. If the authors believe Anderson 2014’s explanation was complete and should not be expressed in new language, then they should set it within quote marks and include a citation. Similarly, text from Anderson 2017 was repeated verbatim. While this text was correctly cited, the text should have been set in quote marks to reflect the fact that this is a quote and not the submitting authors’ original language. This of course, does not mean the results necessarily need to be questioned, but it made me feel uneasy.

 In general, I think that the study would benefit from a greater number of citations from more recent works. Examples the authors may wish to add include Catz et al., 2020, a dental microwear study in ice age Reindeer Ref:1. Some greater degree of citation and discussion of the Younger Dryas climate instability (mentioned by name only once in the submitting authors’ conclusion) is appropriate, especially given climate instability might cause plastic behavioral responses that decouple a hypothesized form-functional relationship between reindeer foot bones and mobility behaviors. Yannic et al., 2020 Ref:2 could be cited with respect to population structure and admixture in North American Caribou populations, while Martino et al., 2024 Ref:3 is an example of a recent morphometric study of metapodial bones in giraffidae. In particular, citing Martino could help demonstrate why foot bones in are especially interesting and why the chosen study approach should be expected to be informative and help clarify the submitting authors’ ambitions.

**Is the study design appropriate and the work technically sound?**

**Response: Partly**

 I mentioned earlier that substantial population structure exists in North American Caribou. The eastern-Canadian and the Beringian lineages have a deep divergence and this may have provided them with the opportunity to adapt over a relatively long time scale to distinct environments which require different mobility behaviors. Morphological differences might have accumulated as a result of selection, but could have also emerged as a result of random drift or because of lurking variables that have not been investigated. Contemporary North American Caribou populations’ differences in mobility are strongly associated with their lineage, and there is admixture between the two populations in the western Canadian population. It is therefore conceivable that morphological differences between migratory and sedentary North American Caribou populations result from their different ancestries and that, even if there is a mechanistic role for morphological differences, that European ice age reindeer may not have evolved analogues of these adaptations because their gene pool did not contain admixture from the eastern Canadian population.

 Moreover, there is an acknowledged semantic difference between observed mobility behaviors in North America and the types of nomadic behavior that other researchers describe in Magdalenian France. So the study design is unable to promise that differences between North American Caribou depend mechanistically on morphology and that those morphological differences should exist in ice age European populations. Rather, an additional study is necessary to determine if this is true.

 That said, if all of this is made clear, the authors can present the case that their study is a good attempt to assess form-functional relationships with the data currently available to them, and identify the above questions as avenues of future study.

**Are sufficient details of methods and analysis provided to allow replication by others?**

**Response:** **Partly**

 See my detailed comments in response to methods.

**Are all the source data and materials underlying the results available?**

**Response: Yes**

 I checked the submitting authors’ Zenodo deposition and the original images, environmental and behavioral classifications are available there. The authors have hence met the criteria. The image files are large (0.725 GB), and it would benefit other researchers if the derived landmark data, which would be much easier to download, were also available (I hope that I did not overlook them if they were present and apologize if I did). I found at least some of the original code and scripts in the PERMANOVA pdf deposits, and it appears these were introduced at the request of a previous reviewer. I personally believe these results better support alternative interpretations to those advocated by the submitting authors, but reserve these comments for my appraisal of their conclusions.

**If applicable, is the statistical analysis and its interpretation appropriate?**

**Response: Partly**

 The authors justify limitations in sample collection from endangered populations, and I am satisfied by that.

 The submitting authors’ statement that there is ‘no measurement error in the data’ is not credible, and it is unclear what is meant by the statement ‘a Procrustes ANOVA was carried out to test the error’. I think what they meant to state is that a partial Generalized Least Squares model found that individual specimen identity was a strong predictor of cross section geometry and that the day of measurement was not, implying a strong reproducibility of measurements. This is not equivalent to zero error; even a scenario where measurements were invariant would not show that. Imagine if I incorrectly measured a bone to have a length of 0 centimeters twice. The fact that my measurements are exactly concordant is not ipso facto a demonstration that I made no mistake. I agree with the authors that it suggests their data probably are not seriously contaminated by error, but suggesting there ‘is no error’ belies the meaning of their analysis.

 With regard to the authors’ methods, using a PERMANOVA approach to explore differences in cross-sectional geometry:

 The authors could have stacked the cross sections into a 3-dimensional matrix for these analyses, to assess full bone shape simultaneously. This approach would have reduced the probability of false positives, because fewer repeated tests are carried out (if we had carried out tests on 20 different cross sections, we would expect one of them would probably yield a significant result at an alpha value of 0.05 for instance), and would have accommodated the possibility that subtle shape changes distinguish migratory or sedentary behaviors that are only evident in full 3-dimensional shape.

 Approaches exist to accommodate this kind of statistical test in the R geomorph package, for example the ProcD.lm() function.

 I note in Table 1 that Caribou subspecies in the geometric study are associated solely with a single mobility pattern and with a single habitat type, and habitat is strongly correlated with mobility pattern. PERMANOVA models may therefore struggle to distinguish whether Caribou ancestry or habitat type have independent contributions to structure. The impression I get is that the dominant signal in the dataset is actually going to be the degree of Euro-Beringian ancestry subspecies possess (indeed, subspecies was found to almost always explain more variety between specimens than mobility type), and that this is strongly regressed upon mobility pattern because mountain and boreal forest populations are almost all described as sedentary and have all experienced genetic admixture from a divergent eastern Canadian population. I do not understand why only PERMANOVA results for mobility group are tabulated in the manuscript body, the three-way PERMANOVA in the Zenodo deposition was essential to complete my review.

 The sentence ‘Finally, once the dependent variables were tested, mobility pattern emerged as a good predictor and, due to the size of the reference sample, we considered it a suitable predictor for discriminant analyses.’ is opaque and needs to be explained in more detail.

 I note that there is some subjective language such as ‘best’ to describe retrieved patterns in PERMANOVA; it is sufficient to state that the p-values were lowest.

 I am confused that explained variances were not presented alongside p-values in the manuscript body. A variable that explains a greater degree of variance might be preferred over a variable that explains very little variance even if the p-value for the second variable is lower, for example (indeed this is the case with ancestry, which is almost universally a superior description of variety, even though it was included as the last sequential term in the PERMANOVA models).

 Where the authors describe their implementation of PERMANOVA: ‘three-way PERMANOVA using the Vegan script in the Adonis package in R-Studio 1.3’, I think this should read ‘adonis function of the Vegan Package’. The documentation of the adonis function shows that it is designed to process distance matrices as input, and was written with the expectation that these would represent species abundances. This may not affect the authors’ results, but procD.lm() of geomorph would have been a more appropriate choice of statistical method, because it is directly designed for 3D landmark data. ProcD.lm performs a partial Generalized Least Squares fit explaining the differences between landmark constellations (or any matrix of continuous data) as a function of independent variables represented in a design matrix, and can be specified to explore sequential, hierarchical or sum of squares methods. Marginal sum of squares would be the most conservative choice, because it would directly test whether the differences between landmark constellations are uniquely explained by mobility in a way that neither habitat or subspecies identity explains. My suspicion is that subspecies identity would explain the majority of morphological difference and that it may not be possible to discern an effect of mobility, when this structure is implemented.

 I am not directly familiar with the syntax for adonis PERMANOVA models, but the structure in the submitting authors’ script implies that they specified their model as interactions between all terms. Most reported model results do not include an interaction. Interpreting Table 1 and 2, I think this is because it is rare that habitat and mobility pattern cross in the heterogeneous sampling across bones. I note that it appears pattern and subspecies never cross, which means that pattern is entirely a proxy of subspecies.

 I note that a previous author requested that sex be investigated. Can the submitting authors justify the omission of sex in their PERMANOVA analyses? I am assuming that all specimens were from mature adult animals, but found no statement to this effect in the manuscript. I apologize if I failed to find such a statement.

**Are the conclusions drawn adequately supported by the results?**

**Response: No**

 Having read the submitting authors’ manuscript in detail, I believe that it is possible that their conclusions could be supported by their analysis. However, I think they actually presented stronger evidence that patterns of admixture between two deeply divergent Caribou source populations are associated with minor skeletal differences between populations with different ancestral affinities.

 When I reviewed the original 3-way PERMANOVA results, which are available with reported R-squared values in full in a supporting PDF on the dedicated Zenodo deposition, I found that the variable which explained the greatest variety in the dependent dataset was subspecies identity, and that this was on the order of twice the value that mobility pattern explained.

 Moreover, terms were added sequentially; mobility pattern was added first while subspecies was added last. The reverse would have been more logical if the authors sought to determine whether mobility explained any variety in osteology that cannot be explained by subsepecies identity or local habitat properties.

 I therefore do not yet personally believe that mobility explains morphological variety in North American Caribou distal limb bone cross-section or that there is a strong reason provided here to justify that the differences found between different North American subspecies should be analogous to any mobility-dependent osteological differences in Reindeer of the European Magdalenian.

 I have therefore concluded that a re-ordered model specification, or use of marginal probabilities is necessary before it can be concluded that a unique morphometric signal of mobility has been identified with scientific validity. Previous reviewer comments about sex and population age structure must be addressed to confirm they do not confound the results.

If applicable, is the statistical analysis and its interpretation appropriate?

Partly

Is the study design appropriate and is the work technically sound?

Partly

Is the work clearly and accurately presented and does it engage with the current literature?

Partly

Are the conclusions drawn adequately supported by the results?

No

Are sufficient details of methods and analysis provided to allow replication by others?

Partly

Are all the source data and materials underlying the results available?

Yes

Reviewer Expertise:

Vertebrate evolution, geometric morphometrics, statistical methods in phylogenetic comparative analyses

I confirm that I have read this submission and believe that I have an appropriate level of expertise to state that I do not consider it to be of an acceptable scientific standard, for reasons outlined above.

## References

[1] : Reindeer feeding ecology and hunting strategies by Magdalenians from Pincevent (Paris Basin, France): New insights from dental microwear textural analyses. *International Journal of Osteoarchaeology* .2020;30(4) : 10.1002/oa.2879 519-528 10.1002/oa.2879

[2] : Harnessing paleo-environmental modeling and genetic data to predict intraspecific genetic structure. *Evol Appl* .2020;13(6) : 10.1111/eva.12986 1526-1542 10.1111/eva.12986 32684974 PMC7359836

[3] : Disentangling morphological variation in metapodials of giraffids: Modern and traditional approaches. *Organisms Diversity & Evolution* .2024;24(2) : 10.1007/s13127-024-00647-w 201-213 10.1007/s13127-024-00647-w

[4] : Permutational Multivariate Analysis of Variance (PERMANOVA ).2014; 10.1002/9781118445112.stat07841 1-15 10.1002/9781118445112.stat07841

# Reviewer report: Reviewer response for version 2

**DOI:** 10.21956/openreseurope.21815.r53329

**Published:** 2025-04-24

**Authors:** María Isabel Borao-Álvarez

**Affiliations:** 1 University of Valencia, Valencia, Valencian Community, Spain

I would like to express my sincere gratitude to the editorial team of *Open Research Europe* and to the authors for the opportunity to review the manuscript entitled *“The ecomorphology of Caribou (Rangifer tarandus): a geometric morphometric study.”*

 This study sets out to establish a robust reference framework of morphometric data based on metacarpals, metatarsals, and phalanges from Canadian caribou populations inhabiting distinct biomes. These populations display varying mobility patterns—either migratory or sedentary—correlated with their ecological settings (boreal forest, mountainous regions, or Arctic tundra).

 The morphometric differences identified between high-mobility populations (typically associated with tundra and mountainous habitats) and those with restricted movement (inhabiting more closed environments) hold significant potential for reconstructing caribou mobility patterns during the Upper Palaeolithic. Such reconstructions offer valuable insights into human subsistence strategies, particularly in regions where caribou/reindeer played a central role in hunter-gatherer economies.

 The authors demonstrate a thorough command of caribou ecology, genetics, behaviour, and morphology. The study employs a rigorous and up-to-date methodological framework, incorporating geometric morphometrics and statistical analysis implemented in the R programming language.

 The digital acquisition and processing of bone scans are appropriately conducted and validated, ensuring the reliability of the landmark-based morphometric data. The statistical procedures are correctly applied and thoughtfully interpreted.

 Moreover, the manuscript is clearly written and logically structured, facilitating a smooth and coherent reading experience.

 In conclusion, I find this to be a well-executed and scientifically valuable contribution. I recommend the manuscript for indexing without reservation and commend the authors for their exemplary work, which represents a significant advancement in the study of prehistoric reindeer hunting strategies.

If applicable, is the statistical analysis and its interpretation appropriate?

Yes

Is the study design appropriate and is the work technically sound?

Yes

Is the work clearly and accurately presented and does it engage with the current literature?

Yes

Are the conclusions drawn adequately supported by the results?

Yes

Are sufficient details of methods and analysis provided to allow replication by others?

Yes

Are all the source data and materials underlying the results available?

Yes

Reviewer Expertise:

My research focuses on reindeer and red deer osseous technology, with particular attention to the acquisition of raw materials and its relation to hunting strategies.

I confirm that I have read this submission and believe that I have an appropriate level of expertise to confirm that it is of an acceptable scientific standard.

# Reviewer report: Reviewer response for version 1

**DOI:** 10.21956/openreseurope.14861.r44843

**Published:** 2024-10-17

**Authors:** Iván Rey-Rodríguez

**Affiliations:** 1 Centro de Investigación Mariña (CIM), MAPAS lab, University of Vigo (Ringgold ID: 16784), Vigo, Galicia, Spain

In my review of the article, I offer several points of feedback to enhance its clarity and focus. Below is a summary of my suggestions and observations regarding different sections of the paper:

 In the abstract I recommend providing a chronological range for the Paleolithic period to help readers unfamiliar with *Rangifer tarandus* better understand the timeframe. Specifically, it would be helpful to clarify whether the study focuses. This would provide important context for readers who may not be specialists in this.

 It is also important to clarify which species the ethological data is based on and whether human influence is considered. This will help frame the study within a relevant biological context, especially for readers interested in how human interactions with reindeer or caribou might have influenced their mobility and bone structure.

 In the title of the project, “Reconstructing habitat type and mobility patterns of *Rangifer tarandus* during the Late Pleistocene in Southwestern France: an ecomorphological study,” the quotation marks should be properly closed, and the species name *Rangifer tarandus* should be italicized to follow standard scientific conventions.

 In Table 1, it would be very useful to include the provenance of the sample and to indicate from which museum the samples were obtained. Additionally, I suggest incorporating a map that shows the current distribution of the caribou populations studied. This would provide a valuable visual aid and help readers better understand the geographic context of the data.

 Clarification is needed on why only specific bone sections were analyzed rather than the complete bone. This methodological decision should be justified, especially considering the potential importance of analyzing the whole structure in morphological studies.

 One question is whether there are any differences between the species in terms of age and sex. Were these factors considered in the study? Furthermore, the Zenodo repository differentiates between right and left bones. Was laterality considered in the analysis?

 The PCA and LDA analyses are well executed, but it would be very interesting to see a visualization of the different morphologies. Plotting these morphologies would make it easier to see the distinctions between the species and the groups being analyzed. This would also strengthen the presentation of the results and make the differences between migratory and sedentary herds more apparent.

 The results are particularly compelling, as they show that mobility, which is linked to habitat, affects the shape of the lower limb bones. This relationship allows for the distinction between migratory and sedentary species. However, since the study is focused on extant species, the archaeological inferences are limited in this article.

 One commendable aspect of the study is the sharing of the CT scans, which adds transparency to the research process. However, it would be ideal if the authors could also include the R script used for their analysis (they indicate them, but it would be better to have them at zenodo), making it easier for other researchers to replicate the study and build upon its findings.

 In summary, with a few adjustments and clarifications, this study has the potential to make a significant contribution to understanding the relationship between habitat, mobility, and bone morphology in *Rangifer tarandus*. I would like to congratulate the authors on this insightful and valuable contribution. Given that this article was published in 2021, and considering the authors' impressive scientific track record, it is clear that this study lays the groundwork for future research in this field. The methodologies and findings presented here establish a solid foundation for continued exploration into the relationship between mobility, habitat, and bone morphology in *Rangifer tarandus*, and I look forward to seeing how this research evolves and expands in the coming years

If applicable, is the statistical analysis and its interpretation appropriate?

Yes

Is the study design appropriate and is the work technically sound?

Yes

Is the work clearly and accurately presented and does it engage with the current literature?

Yes

Are the conclusions drawn adequately supported by the results?

Yes

Are sufficient details of methods and analysis provided to allow replication by others?

Yes

Are all the source data and materials underlying the results available?

Yes

Reviewer Expertise:

paleontology, paleoclimate, geometric morphometrics, rodents, prehistory,

I confirm that I have read this submission and believe that I have an appropriate level of expertise to confirm that it is of an acceptable scientific standard.

# Reviewer report: Reviewer response for version 1

**DOI:** 10.21956/openreseurope.14861.r28842

**Published:** 2022-03-22

**Authors:** Pamela Groves

**Affiliations:** 1 Institute of Arctic Biology, University of Alaska Fairbanks, Fairbanks, AK, USA

I am a little confused by this article as the Abstract and Introduction focus on paleo reindeer in France, but the bulk of the rest is about Canadian caribou and distinguishing between migratory and sedentary herds based on analysis of scanned bones. I understand this paper is setting the groundwork for more research on paleo-French caribou, but that should be in the discussion and the first part of this paper should focus on what it is really about.

 The background on Canadian caribou is possibly more detailed than is needed for this study. That said, it would be useful to have some maps showing the ranges of the populations studied with their migration routes indicated. I would also like earlier in the paper some indication of what is meant by long and short migration distances.

 I don’t feel qualified to assess the statistical methods used in this study and trust there are others with those capabilities. I do wonder though about the bones that were sampled. Is the sex and age of the animals known? Presumably, the bones of an older animal would show more evidence of long migrations or lack there-of. Do larger males put more stress on bones that would contribute to changes? Along those lines, it would be useful to include a brief physiological description of the processes that would contribute to changes in bones based on activity. Finally, are the bones all from the same time period? In the actual text, it would be useful to include an image of at least one scanned bone cross-section.

 When time periods are mentioned, such as Magdalenian or Late Glacial, the timeframe in years should be included. There are a few places with some awkward wording that should be corrected.

 I think if this article is re-framed as a morphometric study of Canadian caribou, it would be a worthwhile contribution to the field. The ability to distinguish between migratory and non-migratory animals within a species does have significant implications for wildlife management and conservation.

If applicable, is the statistical analysis and its interpretation appropriate?

Yes

Is the study design appropriate and is the work technically sound?

Partly

Is the work clearly and accurately presented and does it engage with the current literature?

Partly

Are the conclusions drawn adequately supported by the results?

Partly

Are sufficient details of methods and analysis provided to allow replication by others?

Yes

Are all the source data and materials underlying the results available?

Yes

Reviewer Expertise:

History of paleo megafauna in Alaska. DNA studies of Alaskan caribou and muskoxen.

I confirm that I have read this submission and believe that I have an appropriate level of expertise to confirm that it is of an acceptable scientific standard, however I have significant reservations, as outlined above.
